# All-Cause and Cancer Mortality Trends in Macheng, China (1984–2013): An Age-Period-Cohort Analysis

**DOI:** 10.3390/ijerph15102068

**Published:** 2018-09-20

**Authors:** Chunhui Li, Songbo Hu, Chuanhua Yu

**Affiliations:** 1 Key Laboratory of Environment and Health (HUST), Ministry of Education & Ministry of Environmental Protection, School of Public Health, Tongji Medical College, Huazhong University of Science and Technology, Wuhan 430030, China; chli0201@163.com; 2 School of Health Sciences, Global Health Institute, Wuhan University, Wuhan 430071, China; husbo0910@163.com; 3 School of Public Health, Nanchang University, Nanchang 330019, China

**Keywords:** all-cause mortality, cancer mortality, age-period-cohort model, intrinsic estimator algorithm

## Abstract

The aim was to study the variation trends of all-cause and cancer mortality during 1984–2013 in Macheng City, China. The mortality data were collected from Macheng City disease surveillance points system and Hubei Center for Disease Control and Prevention. The model life table system was used to adjust mortality rates due to an under-reporting problem. An age-period-cohort model and intrinsic estimator algorithm were used to estimate the age effect, period effect, and cohort effect of all-cause mortality and cancer mortality for males and females. Age effect of all-cause mortality for both sexes increased with age, while the age effect of cancer mortality for both sexes reached a peak at the age group of 55–59 years old and then decreased. The relative risks (RRs) of all-cause mortality for males and females declined with the period and decreased by 51.13% and 63.27% during the whole study period, respectively. Furthermore, the period effect of cancer mortality in both sexes decreased at first and then increased. The cohort effect of all-cause and cancer mortality for both sexes born after 1904 presented the pattern of “rise first and then fall,” and decreased by 82.18% and 90.77% from cohort 1904–1908 to 1989–1993, respectively; especially, the risk of all-cause and cancer mortality for both sexes born before 1949 was much higher than that for those born after 1949.

## 1. Introduction

As reported by the World Health Organization, cancer is the second leading cause of death around the world, and approximately 70% of deaths from cancer occur in low- and middle-income countries [1]. Cancer has greatly increased the global burden of disease [2]. Some studies also reported that the cancer mortality appeared to increase in developing countries [3,4,5]. The results from GLOBOCAN 2012 indicated that there were an estimated 8.2 million deaths from cancer in 2012 worldwide, while in China, there were an estimated 2.2 million deaths from cancer (male 1.4 million, female 0.8 million) which accounts for about 1/4 of the total cancer deaths [6]. Furthermore, the cancer mortality and the proportion of the younger patients presented an upward trend during the past 30 years in China [7].

The Chinese government has implemented a series of cancer prevention and control strategies since the 1980s, including improvement of cancer surveillance system, establishment of death-surveillance points, promoting of health education and behavioral interventions, as well as early screening, diagnosis, and treatment. The trend of all-cause or cancer mortality can reflect the health status of residents in a country or region, assess the cost-effectiveness of interventions, and guide the formulation of health policies. 

The city of Macheng, located in the northeast of Hubei Province, is one of the earliest death-surveillance points in China. Due to the development of medical care and the intervention of public health, the all-cause mortality in 2000s approximately decreased one-third compared to that in 1970s. However, the total cancer mortality increased with a rate of averaging 2.01% [8,9,10]. Therefore, quantitative research is helpful to reflect the true trends of cancer and assess the impact of intervention measures and potential risk factors. 

Numerous studies focused on the trend analyses of some specific cancer mortality using an age-period-cohort (APC) model in China [11,12,13]. The mortality risk can be decomposed into age effects, period effects, and cohort effects using the APC model. Age effects are varying risks associated with chronological age groups; period effects are variations over time periods or calendar years that influence all age groups simultaneously; cohort effects are changes across groups of individuals born in the same year or years. Although limited research has reported the total cancer mortality [14,15], they failed to analyze the cohort effect of the cancer mortality risk. Wang et al. [16] researched the trend of cancer mortality in rural China during 1990–2010; however, the population was limited to the rural environment. In this paper, we analyzed the trend of all-cause and cancer mortality in Macheng City and discussed the age effect, period effect, and cohort effect using an APC model. The results could assist in exploring the impacts of potential factors (social economy, lifestyle, behavior, and medical treatments) on the health of residents.

## 2. Materials and Methods 

### 2.1. Data Source

The age- and sex-specific population, and all-cause and cancer mortality were collected from Macheng City disease surveillance points (DSPs) system and Hubei Center for Disease Control and Prevention. The Macheng DSP system was established in the early 1980s. The DSPs system has been used for decades to provide mortality data on health-care decision-making and performance evaluation, and the data quality is improved with the continued perfection of disease surveillance standard guidelines and the reporting system [17]. For deaths in hospitals, doctors certified the cause of death, while for death outside hospitals, village health workers, or township or community hospital staff complete a death certificate based on a verbal autopsy. As the smaller proportion of deaths in urban areas occurred at home than that in rural areas, quality of surveillance is better in urban than in rural areas [18]. In addition, the under-reporting rate is estimated every three years by a survey that covers 5% of the surveillance population, and rural areas have a higher under-reporting rate than urban areas [19]. 

In this study, the mortality data we used were from the age groups 20–24 years to 80–84 years old due to the lower death from cancer under 20 years old and the aggregated death statistics above 85 years old. The time horizon of data started from 1984–1988 and ended with 2009–2013 (with 5 years per period). The mortality rates were computed directly from observed deaths and average population counts. Due to the under-reporting problem, the model life table systems including the Coale–Demeny model life table system and the United Nations model life table system were used to adjust mortality rates.

### 2.2. Statistical Analysis 

The APC Model, a statistical method based on the Poisson distribution, is widely applied in epidemiologic, demographical, and sociological fields, which can be used to extract information from cross-sectional data regarding changes of socioeconomic, environment, and lifestyle in the morbidity and mortality risk [20], termed as cohort effect. The intrinsic estimator (IE) algorithm proposed by Fu [21] was used to estimate the parameters in the APC model by orthogonal decomposition of the parameter space, that is, age effects, period effects, and cohort effects. The IE has been confirmed as an estimable, unbiased, and asymptotic estimator [22,23]. The relative risk (RR) was calculated to interpret the age effects, period effects, and cohort effects more intuitively. Fitting deviance, the Akaike information criterion (AIC), and the Bayesian information criterion (BIC) enabled the evaluation of the goodness-of-fit of the APC model. All the analyses were performed using R 3.43 and Stata 14.0 (StataCrop. LP, College Station, TX, USA).

## 3. Results

### 3.1. Descriptive Analysis of All-Cause and Cancer Mortality Trends

**Figure 1:**
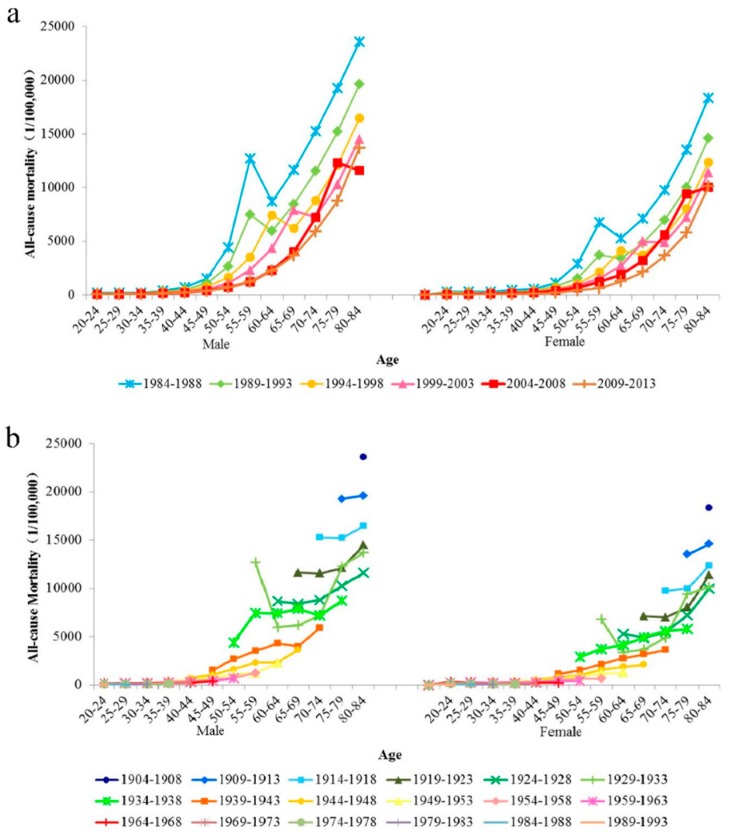
Age-specific all-cause mortality rates of Macheng City residents, male and female, 1984–2013. (**a**) The age-specific all-cause mortality by period. (**b**) The age-specific all-cause mortality by birth cohort.

The all-cause deaths during 1984–2013 in Macheng City presented a slight decrease, while the cancer deaths had a 74% increase and the proportion of cancer deaths continued to rise; for age-specific deaths, the age of cancer deaths tended towards younger people (Appendix A). The trends of age-specific all-cause mortality for males and females during 1984–2013 are shown in Figure 1. Figure 1a,b show the age-specific all-cause mortality by period and cohort, respectively. The all-cause mortality for both sexes reached a peak after 55 years old for each period except for 2009–2013. However, the all-cause mortality of males in all age groups was higher than that of females from 1984 to 2013. The age patterns in Figure 1a,b showed similar trends. The younger birth cohort showed a lower all-cause mortality for both sexes.

**Figure 2:**
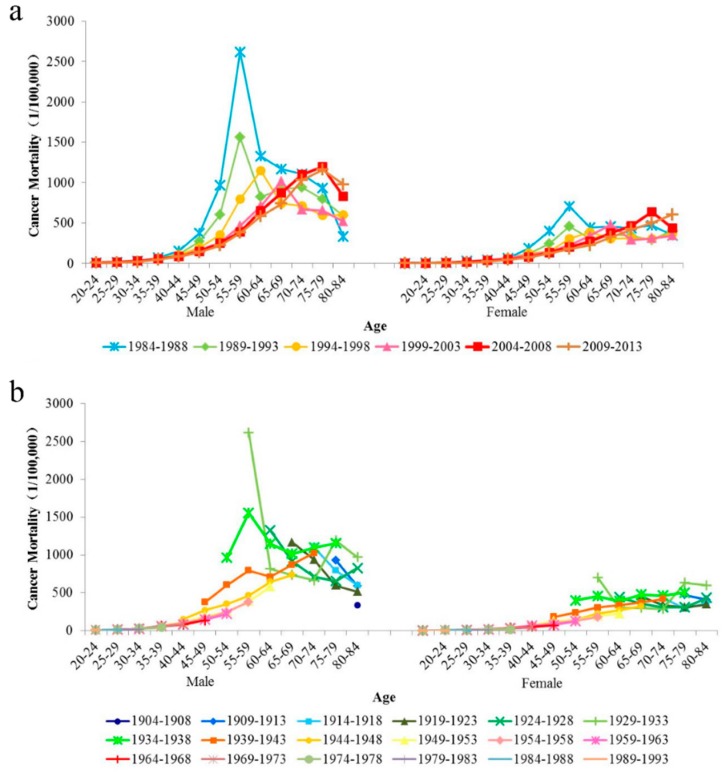
Age-specific cancer mortality rates of Macheng City residents, male and female, 1984–2013. (**a**) The age-specific cancer mortality by period. (**b**) The age-specific cancer mortality by birth cohort.

Figure 2 indicates the trends of age-specific cancer mortality for males and females during 1984–2013 by period (Figure 2a) and cohort (Figure 2b). Given a period, the cancer mortality for both sexes generally increased at first and then decreased with age. Similarly, males had a higher cancer mortality than females from 1984–2013. The variation trend of cancer mortality for both sexes by birth cohort showed that the cancer mortality decreased with age for some birth cohorts. 

However, we cannot distinguish the contributions of age, period, and cohort on mortality risk, that is, age effect, period effect, and birth cohort effect. For a given age group, the period effect and cohort effect of mortality risk are confounded. Thus, it is necessary to analyze the age effect, period effect, and cohort effect and provide the accurate quantitative assessments using the APC model [24,25]. 

### 3.2. Age, Period, and Birth Cohort Effects of All-Cause Mortality 

**Figure 3:**
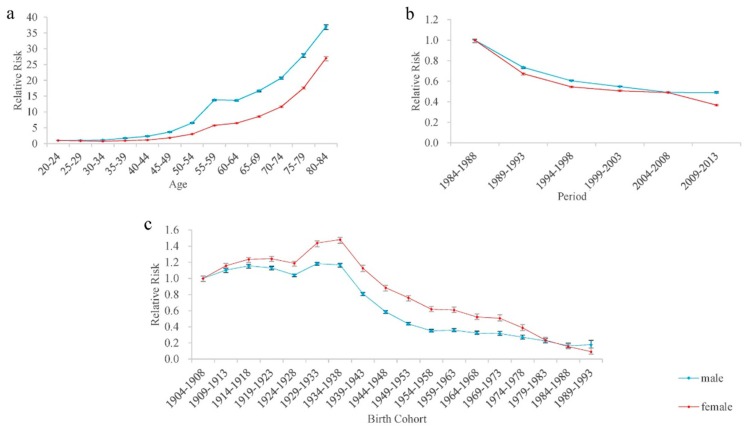
The relative risk and 95% confidence intervals of age, period, and cohort effects of all-cause mortality risk among Macheng City residents, 1984–2013. (**a**) The relative risk and 95% confidence intervals of age effects of all-cause mortality risk. (**b**) The relative risk and 95% confidence intervals of period effects of all-cause mortality risk. (**c**) The relative risk and 95% confidence intervals of birth cohort effects of all-cause mortality risk.

The results of the APC model analysis of all-cause mortality are shown in Table 1 and Figure 3. According to the results (Figure 3c), the age effect of all-cause mortality for both sexes essentially displayed a rising trend with age. Using the group of aged 20–24 years old as a reference, the RRs of the 80- to 84-year-old group were the highest, i.e., 37.01 and 27.05, respectively for males and females. Overall, males had a higher risk of all-cause mortality than females in all age groups.

The estimated period effect RRs of all-cause mortality displayed similar monotonic decreasing trends for males and females, and females had a larger reduction of the RRs than males during 1984–2013 (Figure 3b). Compared with period group 1984–1988, the RRs of all-cause mortality in period group 2009–2013 for males and females were 0.49 and 0.37, respectively. 

The cohort effect RRs of all-cause mortality for males and females in Figure 3c presented a “rise first and then fall” pattern from birth cohort 1904–1908 to birth cohort 1989–1993; however, the risk of females was higher than that of males in all cohorts. Using birth cohort 1904–1908 as a reference, the highest risk of all-cause mortality for males and females was in birth cohort 1929–1933 (RRs = 1.18) and birth cohort 1934–1938 (RRs = 1.48), respectively. Compared with the oldest birth cohort 1904–1908, the cohort RRs of all-cause mortality for males and females in the youngest birth cohort 1989–1993 decreased by 82.18% and 90.77%, respectively.

### 3.3. Age, Period, and Birth Cohort Effects of Cancer Mortality

**Figure 4:**
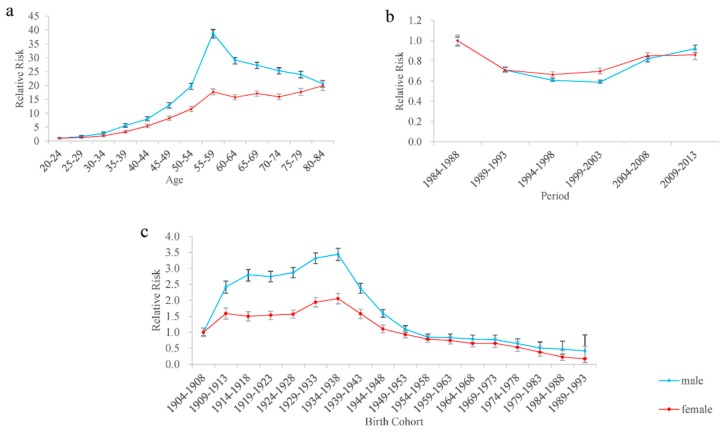
The relative risk and 95% confidence intervals of age, period, and cohort effects of cancer mortality risk among Macheng City residents, 1984–2013. (**a**) The relative risk and 95% confidence intervals of age effects of cancer mortality risk. (**b**) The relative risk and 95% confidence intervals of period effects of cancer mortality risk. (**c**) The relative risk and 95% confidence intervals of birth cohort effects of cancer mortality risk.

The estimated age effects, period effects, cohort effects and RRs of cancer mortality for both sexes are shown in Table 1 and Figure 4, respectively. The age RRs of cancer mortality for males reached a peak in the group aged 55–59 years old and then decreased with age, while the RRs for females generally increased with age (Figure 4a). Compared with the group of subjects aged 20–24 years old, the age RRs of 55- to 59-year-old group of cancer mortality for males and females were 38.78 and 17.69, respectively. In general, the age RRs of cancer mortality for males were higher than that for females in all age groups. 

The period RRs of cancer mortality for both sexes decreased and then increased during the whole study period. Compared to the period 1984–1988, the RRs of the lowest risk of cancer mortality for males (period 1999–2003) and females (period 1994–1998) were 0.59 and 0.67, respectively. The cohort RRs of cancer mortality for males and females in Figure 4c showed similar trends to that of all-cause mortality, except that males had higher cohort RRs of cancer mortality than females. The RRs of cancer mortality for males and females reduced by 58.04% and 82.95% from birth cohort 1904–1908 to 1989–1993, respectively. 

## 4. Discussion

We analyzed the long-term trends of all-cause and cancer mortality in Macheng City and estimated their age effects, period effects, and cohort effects using the APC model. The results showed that all-cause mortality for each age group in both sexes decreased with period (except for period 2004–2008) and birth cohort (except for birth cohort 1929–1933), while the variations in cancer mortality over the period and birth cohort for each age group presented different trends, especially the cancer mortality rates in older groups increased with period and cohort. It indicated the potential effects of period and cohort on the trends of all-cause and cancer mortality. The mortality risk was decomposed into age effect, period effect, and cohort effect by APC model to interpret the effect of risk factors related to period and cohort effect on mortality more directly. Here, we discuss the potential risk factors associated with age, period, and cohort effects of all-cause and cancer mortality, that is, macro-level factors.

Age is one of the important factors for the all-cause and cancer death. According to our results, the age effect of all-cause and cancer mortality risk for both sexes in Macheng basically showed an increased trend with age, which indicated that the higher risk of all-cause and cancer mortality was observed in elderly people. This kind of trend is mainly attributed to more and more serious aging of the population in China [26]. The elderly people were more vulnerable to the effects of illness, injuries and environmental pollution than younger people [27,28]. Furthermore, males had equal or higher RRs of all-cause and cancer mortality than females in our study. It is speculated that physiological differences between sexes may be a factor [29], but further studies are needed. 

Given the period effect of all-cause mortality, there was a net decrease of 51.13% and 63.27% for males and females in the period from 1984–1988 to that from 2009–2013, indicating an annual average negative growth of 1.70% and 2.11%, respectively. The decreased trend of the period effect of all-cause mortality was consistent with other studies [30,31]. The underlying reasons for the decreasing trend was likely to be the rapid urbanization which improved medical technology and increased health awareness, health care resources, and public health infrastructure investment [32]. However, the period effect of cancer mortality for males and females decreased first, and then increased in the period from 2004–2008 and in the period from 1999–2003, respectively. According to the existing literature [33,34], population health is improving; however, a shift from communicable diseases to chronic non-communicable diseases changed the epidemiological patterns due to the rapid socioeconomic development and the increasing demographic status. China has experienced an epidemiological transition (shifting from the infectious diseases and perinatal conditions to chronic diseases and injuries) with an unabated momentum, and cancer has become a major cause of death in China [35,36]. The shift in the causes of death was attributed to improved medical care, advanced living standards, and better nutrition conditions. However, accordingly, behavior risk factors including changing dietary, decreased physical activity, and high tobacco consumption have dramatically increased as societal change progresses [36]. Thus, the decreasing of other-cause mortality (e.g., infectious diseases, maternal diseases, nutritional diseases, and injuries) and increased behavior risk factors may allow the relatively increased cancer mortality risk for Macheng residents aged 20–84 years old after 1999. The unsustainable downward trend of the period effect of cancer mortality indicates that improvement of medical technology and living standards and development of society could not reduce the cancer mortality. Thus, the cohort effect may play an important role in the trends of cancer mortality. 

The cohort effect of the all-cause and cancer mortality for Macheng residents born after 1904 reached their peak in the birth cohort 1934–1938 for both sexes, and then declined; however, the risk of all-cause and cancer mortality overall decreased during the whole study cohort. It has been reported that poor socioeconomic status in childhood was associated with the higher mortality and shorter lifespan [37]. The higher risk of all-cause and cancer mortality was obverted before 1949, which may be explained by the poor socioeconomic status and public health conditions caused by the World War II in China [38]. With the development of the social economy and improvement of health awareness and medical care, the cohort effects of all-cause and cancer mortality for those born after 1949 were much lower than that for those born before 1949 and continued to decrease. Furthermore, an interesting finding was that females had the higher RRs of cohort effects of all-cause mortality than males, while males had the higher RRs of cancer mortality than females. It has been reported that males are prone to develop cancer and have worse overall survival and higher mortality rates [39]. The potential reasons may be that males are more likely to be exposed to risk factors for cancer, such as smoking and alcohol drinking. Another several studies reported that socioeconomic inequalities and lower levels of education are associated with higher all-cause mortality in western Europe countries [40,41]. In China, there was gender inequality in the education system and inequality against girls still exists today [42]. Our study was limited to people of Macheng City. Hence, gender differences in cohort effects of all-cause and cancer mortality will need further study to validate our findings.

In addition, it has been reported that nearly 60% of cancer deaths can be explained by modifiable risk factors, of which tobacco smoking is the predominant contributor in China [43]. China accounts for approximately 40% of the world cigarette consumption [44]. A nationwide smoking epidemiology survey in 2002 indicated that the prevalence of smoking in China was still at a high level, but the smoking cessation rate had increased [45]. Furthermore, the people who smoked tends to be younger, and an estimated 72% of Chinese adolescents who were over 15-year-old had been exposed to tobacco, including those exposed to second-hand smoke [46]. Smoking rates in females who were younger than 25 years old presented an obviously ascending trend [47]. 

Deterioration of the ecological environment may lead to the increasing of cancer mortality [48,49]. Previous studies reported the relationship between environmental pollution and human health and cancer mortality [50,51,52,53]. Liu found that death and cancer mortality rates were positively associated with economic growth and air pollution [50]. Another study proposed an advanced method to improve the accuracy of mortality estimate based on the integrating PM_2.5_ concentrations and the regional air quality model, which indicated that the mortality could be influenced by the air quality [54]. Dietary habit is another risk factor of human health and cancer in China, and responsible for nearly 16% of cancer deaths [43]. A report from the Comparative Risk Assessment Project have indicated that about 6% of cancer deaths could be attributed to low vegetable and fruit intake in middle-income countries [55]. However, a higher population attributable fraction (PAF) for insufficient intake of vegetable and fruit in China accounted for 14% of cancer deaths. With the development of the economy, great changes have taken place in dietary habits. The proportion of animal fat in the diet increased 70.73% from 1985 to 2001 [56]. Therefore, the trends of period effects may be relevant to the change of environment and dietary habits for Macheng residents.

Besides, other risk factors, such as overweight/obesity, physical inactivity, and reproductive factors, also played a role in human health and cancer. A prospective cohort study showed that severe obesity (BMI > 37 kg/m^2^) has a higher mortality [57]. Singh et al. reported that physical inactivity may influence the cancer mortality and incidence [58,59]. The reproductive factors were mainly related to the breast, ovarian, and endometrial cancer [60,61]. In addition, except for controlling the risk factors, the earlier diagnosis of cancer and optimal treatment may have an important role on cancer survival. 

There are some limitations in the present study. Although APC analysis can depict the entire complex of social, historical, and environmental factors that simultaneously affect populations, it focuses on the study of population level instead of individuals. Therefore, relevant speculation from results of the APC analysis still need further validation in the studies at the individual level. 

## 5. Conclusions

In summary, the all-cause mortality in both sexes in Macheng was on a declining trend during 1984–2013, which is close to the variation of period and cohort effects on the all-cause mortality. The cohort effect of cancer mortality for males and females born before 1949 was much higher than those born after 1949, which indicated that the important impact of the social economy and public health condition changes. In addition, the increased period effect of cancer mortality risk from the late 1990s may be explained by the epidemiological transition and increased risk factors for cancer. 

## Figures and Tables

**Table 1:** APC model analysis results of all-cause and cancer mortality in Macheng.

Characteristic	All-Cause				Cancer			
	Male		Female		Male		Female	
	Coef.	95% CI	Coef.	95% CI	Coef.	95% CI	Coef.	95% CI
Age								
20–24	−1.75 ***	(−1.82, −1.67)	−1.21 ***	(−1.27, −1.15)	−2.35 ***	(−2.62, −2.08)	−1.94 ***	(−2.24, −1.65)
25–29	−1.74 ***	(−1.81, −1.67)	−1.36 ***	(−1.42, −1.30)	−1.86 ***	(−2.06, −1.66)	−1.74 ***	(−1.98, −1.49)
30–34	−1.65 ***	(−1.71, −1.58)	−1.48 ***	(−1.54, −1.42)	−1.34 ***	(−1.50, −1.18)	−1.33 ***	(−1.53, −1.13)
35–39	−1.21 ***	(−1.25, −1.15)	−1.27 ***	(−1.33, −1.22)	−0.64 ***	(−0.75, −0.52)	−0.75 ***	(−0.90, −0.60)
40–44	−0.90 ***	(−0.94, −0.85)	−1.06 ***	(−1.11, −1.02)	−0.28 ***	(−0.37, −0.18)	−0.27 ***	(−0.38, −0.15)
45–49	−0.44 ***	(−0.47, −0.41)	−0.60 ***	(−0.63, −0.56)	0.20 ***	(0.12, 0.27)	0.15 **	(0.05, 0.24)
50–54	0.13 ***	(0.10, 0.15)	−0.10 ***	(−0.13, −0.08)	0.63 ***	(0.57, 0.68)	0.49 ***	(0.41, 0.57)
55–59	0.88 ***	(0.86, 0.89)	0.53 ***	(0.51, 0.55)	1.30 ***	(1.26, 1.34)	0.92 ***	(0.86, 0.98)
60–64	0.87 ***	(0.85, 0.88)	0.65 ***	(0.63, 0.67)	1.01 ***	(0.97, 1.05)	0.80 ***	(0.75, 0.86)
65–69	1.07 ***	(1.05, 1.08)	0.93 ***	(0.91, 0.95)	0.95 ***	(0.91, 0.99)	0.89 ***	(0.83, 0.94)
70–74	1.29 ***	(1.27, 1.30)	1.24 ***	(1.22, 1.26)	0.87 ***	(0.83, 0.92)	0.82 ***	(0.75, 0.88)
75–79	1.59 ***	(1.56, 1.60)	1.65 ***	(1.63, 1.67)	0.82 ***	(0.77, 0.87)	0.92 ***	(0.85, 0.99)
80–84	1.87 ***	(1.84, 1.88)	2.08 ***	(2.05, 2.10)	0.67 ***	(0.61, 0.73)	1.04 ***	(0.95, 1.12)
Period								
1984–1988	0.47 ***	(0.45, 0.48)	0.56 ***	(0.54, 0.58)	0.27 ***	(0.23, 0.31)	0.23 ***	(0.18, 0.29)
1989–1993	0.17 ***	(0.15, 0.17)	0.17 ***	(0.15, 0.18)	−0.06 ***	(−0.09, −0.03)	−0.10 ***	(−0.14, −0.06)
1994–1998	−0.03 ***	(−0.03, −0.02)	−0.04 ***	(−0.05, −0.03)	−0.22 ***	(−0.24, −0.19)	−0.17 ***	(−0.20, −0.13)
1999–2003	−0.13 ***	(−0.13, −0.12)	−0.11 ***	(−0.12, −0.10)	−0.25 ***	(−0.27, −0.22)	−0.12 ***	(−0.16, −0.08)
2004–2008	−0.24 ***	(−0.25, −0.22)	−0.14 ***	(−0.16, −0.13)	0.07 ***	(0.04, 0.10)	0.07 **	(0.03, 0.11)
2009–2013	−0.24 ***	(−0.25, −0.22)	−0.43 ***	(−0.45, −0.41)	0.19 ***	(0.15, 0.23)	0.08 **	(0.03, 0.14)
Birth Cohort								
1904–1908	0.64 ***	(0.60, 0.67)	0.42 ***	(0.39, 0.46)	−0.23 ***	(−0.35, −0.11)	0.14 *	(0.00, 0.28)
1909–1913	0.74 ***	(0.71, 0.76)	0.57 ***	(0.54, 0.60)	0.64 ***	(0.56, 0.72)	0.60 ***	(0.49, 0.71)
1914–1918	0.78 ***	(0.76, 0.80)	0.64 ***	(0.61, 0.66)	0.79 ***	(0.72, 0.85)	0.54 ***	(0.45, 0.64)
1919–1923	0.76 ***	(0.74, 0.78)	0.64 ***	(0.62, 0.67)	0.77 ***	(0.71, 0.83)	0.57 ***	(0.48, 0.65)
1924–1928	0.68 ***	(0.66, 0.69)	0.60 ***	(0.57, 0.62)	0.81 ***	(0.76, 0.87)	0.59 ***	(0.51, 0.67)
1929–1933	0.81 ***	(0.79, 0.82)	0.79 ***	(0.76, 0.81)	0.96 ***	(0.91, 1.01)	0.80 ***	(0.73, 0.88)
1934–1938	0.80 ***	(0.77, 0.81)	0.82 ***	(0.79, 0.84)	0.99 ***	(0.94, 1.05)	0.86 ***	(0.78, 0.94)
1939–1943	0.43 ***	(0.40, 0.45)	0.54 ***	(0.51, 0.58)	0.62 ***	(0.56, 0.69)	0.60 ***	(0.50, 0.69)
1944–1948	0.11 ***	(0.07, 0.13)	0.30 ***	(0.26, 0.34)	0.22 ***	(0.15, 0.30)	0.24 ***	(0.13, 0.35)
1949–1953	−0.19 ***	(−0.22, −0.15)	0.15 ***	(0.10, 0.19)	−0.14 **	(−0.23, −0.05)	0.07	(−0.04, 0.20)
1954–1958	−0.41 ***	(−0.45, −0.36)	−0.05	(−0.10, 0.00)	−0.39 ***	(−0.50, −0.29)	−0.09	(−0.23, 0.05)
1959–1963	−0.39 ***	(−0.44, −0.33)	−0.06 *	(−0.12, −0.01)	−0.42 ***	(−0.54, −0.29)	−0.15	(−0.31, 0.01)
1964–1968	−0.49 ***	(−0.54, −0.42)	−0.22 ***	(−0.28, −0.15)	−0.47 ***	(−0.61, −0.33)	−0.28 **	(−0.46, −0.09)
1969–1973	−0.51 ***	(−0.58, −0.43)	−0.25 ***	(−0.32, −0.17)	−0.49 ***	(−0.66, −0.33)	−0.27 *	(−0.49, −0.05)
1974–1978	−0.66 ***	(−0.75, −0.57)	−0.51 ***	(−0.61, −0.42)	−0.67 ***	(−0.88, −0.46)	−0.48 **	(−0.76, −0.20)
1979–1983	−0.86 ***	(−0.97, −0.73)	−1.01 ***	(−1.14, −0.88)	−0.91 ***	(−1.21, −0.60)	−0.82 ***	(−1.21, −0.42)
1984–1988	−1.15 ***	(−1.32, 0.98)	−1.43 ***	(−1.61, −1.24)	−0.98 ***	(−1.39, −0.56)	−1.32 ***	(−1.94, −0.70)
1989–1993	−1.09 ***	(−1.34, −0.82)	−1.95 ***	(−2.34, −1.56)	−1.10 **	(−1.88, −0.32)	−1.62 **	(−2.81, −0.43)
Intercept	7.09 ***	(7.06, 7.11)	6.73 ***	(6.71, 6.76)	5.10 ***	(5.05, 5.16)	4.42 ***	(4.34, 4.51)

*** indicates *p* < 0.001; ** indicates *p* < 0.01; * indicates *p* < 0.05.

## Appendix A

**Table A1:** Age-specific all-cause deaths during 1984–2013 in Macheng City.

Age Group	1984–1988	1989–1993	1994–1998	1999–2003	2004–2008	2009–2013
20–24	1318	1148	691	387	238	257
25–29	828	1067	867	539	298	244
30–34	857	676	780	753	451	287
35–39	951	1047	767	963	867	574
40–44	1096	1063	1186	997	1088	1056
45–49	1503	1612	1558	1667	1356	1528
50–54	2276	2342	2277	2241	2211	1877
55–59	3465	3458	3160	3326	2697	2988
60–64	6157	5642	5099	4872	3928	4131
65–69	6052	5990	5152	5431	4666	5234
70–74	5982	6359	6128	5895	5097	5864
75–79	3038	3483	3347	4372	3666	4939
80–84	2162	2043	2281	2888	2915	3359
Total	35,685	35,930	33,293	34,331	29,478	32,338

**Table A2:** Age-specific cancer deaths during 1984–2013 in Macheng City.

Age Group	1984–1988	1989–1993	1994–1998	1999–2003	2004–2008	2009–2013
20–24	42	46	48	25	29	29
25–29	42	71	69	56	58	37
30–34	88	64	103	131	118	56
35–39	122	156	152	251	273	165
40–44	185	203	265	253	347	380
45–49	311	329	356	413	376	542
50–54	421	468	427	438	606	575
55–59	575	615	611	591	662	894
60–64	776	674	671	723	856	954
65–69	514	569	540	629	798	963
70–74	354	426	432	457	612	888
75–79	125	142	147	233	302	561
80–84	37	59	74	94	166	220
Total	3592	3822	3895	4294	5203	6264

**Table A3:** Goodness-of-fit measurements of APC models.

Index	All-Cause		Cancer	
	Male	Female	Male	Female
Deviance	3708.55	1759.00	764.75	255.84
AIC	57.39	32.15	17.74	10.55
BIC	3516.86	1567.30	573.06	64.15
